# Supplementary material for: Supporting frail older people with depression and anxiety: a qualitative study
Source: Aging Ment Health. 2019 Oct 16;24(12):1977–84. doi: 10.1080/13607863.2019.1647132 (PMC8842711; doi:10.1080/13607863.2019.1647132)
Supplement: Supplemental Material [file CAMH_A_1647132_SM8113.pdf]

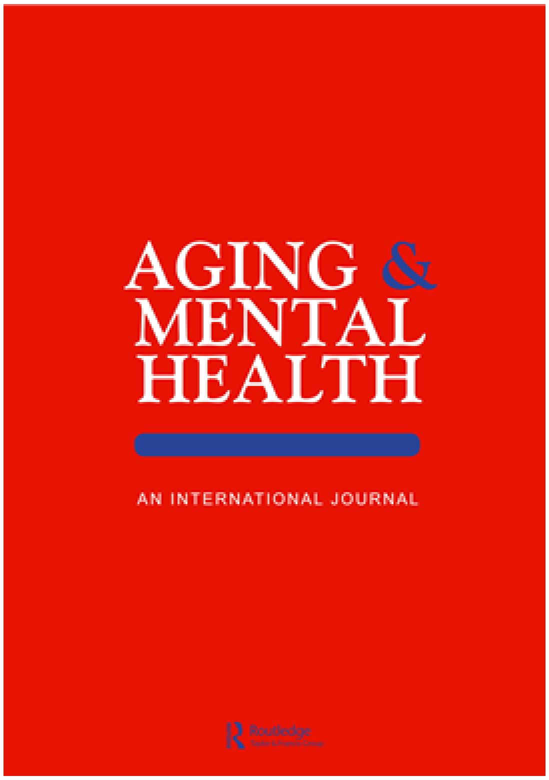

Supporting frail older people with depression and anxiety: a qualitative study

Journal:	*Aging and Mental Health*
Manuscript ID	CAMH-2019-0023.R1
Manuscript Type:	Original Article
Keywords:	Depression < Mood Disorders, Anxiety and Trauma related disorders, Anxiety < Mood Disorders, Anxiety and Trauma related disorders, Qualitative methods, Extreme Old Age, Physical Health Status

SCHOLARONE™
Manuscripts

Supporting frail older people with depression and anxiety: a qualitative study

Abstract

Objectives: Depression and anxiety are common in later life, particularly when people are frail. This leads to reduced quality of life, faster decline in physical health and increased health/social care use. Available treatments are commonly not tailored to people with frailty. We explored frail older peoples' experiences of depression and/or anxiety and how services could be adapted to their needs.

Methods: Semi-structured interviews with 28 older people in the UK purposively sampled for practice location and severity of frailty and anxiety/depression. We asked about symptoms, interactions with physical health, help-seeking and treatments and what might help in future. We audio-recorded and transcribed interviews, using thematic analysis to inductively derive themes.

Results: Frail older people had low expectations of their wellbeing at this point in life due to multiple physical health issues and so anxiety and mild depressive symptoms were normalised. There was a particular reluctance and uncertainty regarding help-seeking for anxiety. Treatments were considered appropriate where they aligned with coping skills developed over their lifetime, and facilitated independence and problem-solving skills. Most older people felt their knowledge of mental health was limited and relied upon information about and endorsement of therapies from an expert. This was usually their GP, but access was often problematic. Online methods of accessing information and therapies were not popular.

For Peer Review Only

Conclusion: Mental health support for frail older people needs to address late-life anxieties as well as depression, account for physical health issues, align with older people's need for independence and facilitate coping skills.

Background

Frailty is present in around 11% of adults aged 65+, but may range from 4.0% to 59.1% depending on the definition used (Collard, Boter, Schoevers, & Oude Voshaar, 2012). It is recognised as a dynamic process involving deficiencies across multiple physiological systems, characterised by reduced functional reserves and vulnerability to adverse health outcomes (Clegg & Hassan-Smith, 2018; Clegg, Young, Iliffe, Rikkert, & Rockwood, 2013). Although it lacks a universal definition, symptoms typically included in frailty models are unintentional weight loss, slow gait speed, muscle weakness, low energy and low physical activity (Fried et al., 2001). Mental health is often overlooked in these definitions, despite psychological resilience being seen as an important part of managing physical frailty (Shaw et al., 2018). Reductions in mobility and independence can strongly impact mood - frail older adults are four times more likely to experience clinically significant anxiety or depression (Ni Mhaolain et al., 2012), with up to half experiencing depressive symptoms (Vaughan, Corbin, & Goveas, 2015). Symptoms of anxiety are less well documented in this population, despite frequently co-existing with depression (Braam et al., 2014). Anxiety and depression are associated with increased rates of functional decline, cognitive decline and healthcare service utilisation (Meeks, Vahia, Lavretsky, Kulkarni, & Jeste, 2011; Wolitzky-Taylor, Castriotta, Lenze, Stanley, & Craske, 2010). In combination with frailty, anxiety further increases mortality rates (Dent & Hoogendijk, 2014), whilst depression is associated with neurocognitive decline (Potter, McQuoid, Whitson, & Steffens, 2016) and use of GP and psychiatric services (Djernes, Gulmann, Foldager, Olesen, & Munk-Jørgensen, 2011).

URL: http:/mc.manuscriptcentral.com/camh
For Peer Review Only

However, depression and anxiety are commonly underdiagnosed in this population (Mitchell, Rao, & Vaze, 2010).

Mental health treatments for frail older adults are currently limited, and mental health symptoms are often given lower priority by healthcare professionals than physical health problems (Rachael Frost, Beattie, Bhanu, Walters, & Ben-Shlomo, 2019). Those in their 80s or 90s with depressive symptoms are more likely to be prescribed antidepressants and substantially less likely to be referred to psychological therapies than those in their late 50s and early 60s (Walters, Falcaro, Freemantle, King, & Ben-Shlomo, 2018). Although antidepressants show effects upon response (>50% reduction in symptoms) in people aged 55+ (Kok, Nolen, & Heeren, 2012), the vast majority of antidepressant trials are in those aged under 75 and exclude people with medical comorbidities (Benraad et al., 2016). Effectiveness of antidepressants is therefore less clear in frailer populations, and taking multiple medications is recognised as a contributor to frailty (Gutiérrez-valencia, Hospitalario, & Irunlarrea, 2018). Many older people prefer talking therapies above medication for depression and anxiety (Gum et al., 2006; Landreville, Landry, Baillargeon, Guérette, & Matteau, 2001; Mohlman, 2011). Cognitive behavioural therapy, life review and problem solving therapy show some positive effects in older people, but for those who are frailer the evidence is limited, mostly focusing upon problem solving therapy (R Frost, Bauernfreund, & Walters, 2018; Gould, Coulson, & Howard, 2012b, 2012a; Kirkham, Choi, & Seitz, 2016; Lan, Xiao, & Chen, 2017). Access to these therapies is still low in the UK, particularly for those aged 75+ (NAPT, 2013).

Previous qualitative studies have mainly focussed upon late life depression, finding that that older people hold negative perceptions of treatments, minimise and normalise depression as part of later life or difficult circumstances and place a strong focus upon personal actions they

For Peer Review Only

can take (Corcoran et al., 2013; Holm & Severinsson, 2014). The small number of qualitative studies carried out in older people with anxiety show that anxiety is also attributed to loss and age-related decline, but that it is more difficult to identify than depression (Kingstone et al., 2017; Knight & Winterbotham, 2019), although information is sought from healthcare professionals, online resources and their own internal coping strategies (Zapata et al., 2018). Most qualitative studies have been carried out in those aged 65-75 with better physical health, focussing upon experiences of depression rather than treatment preferences. We therefore aimed to explore frail older adults' experiences of depression and anxiety, their views regarding help-seeking, and ways in which services could be adapted to better meet their needs.

Methods

We recruited participants using mailouts from five UK general practices across one semi-rural, one suburban and three urban areas who were aged 75+, with frailty and experiencing symptoms or a diagnosis of depression and/or anxiety. People with advanced dementia and <6 months life expectancy were excluded. Practice staff searched their patient records to identify patients classified as moderately to severely frail according to their practice's frailty list (e.g. electronic frailty scale) and aged 75+ years. As depression/anxiety can be under-diagnosed in this population, we asked practices to send a study invitation to a proportion with and without a diagnosis of anxiety and/or depression recorded in their medical records. The mailed invitation letter and information leaflet encouraged people to self-identify using an adapted brief 2-item screening questionnaires in the leaflet (2-item Patient Health Questionnaire and 2-item Generalized Anxiety Disorder scale). Both are originally validated in older populations (Li, Friedman, Conwell, & Fiscella, 2007; Wild et al., 2014), but were edited for brevity and changing the two-week timescale to 'recently'. Practice GPs reviewed

the list of those to approach prior to mail-out and removed people that met the exclusion criteria or they considered inappropriate to contact. Interested respondents were screened by researchers over the telephone using the same adapted questions as the leaflet to confirm symptoms and were asked about functional difficulties (e.g. reduced mobility) to confirm frailty.

We conducted semi-structured face-to-face interviews, mainly at participants' homes (or another convenient location if requested), lasting approximately one hour (range 34-89 min). Interviews were conducted by RF (n=19, a health services researcher) or PN (n=9, an academic GP, not disclosed to interviewees). Each observed 1-2 of the other's interviews to ensure consistency. Topics included a typical week; historical and current experiences of depression and anxiety; coping mechanisms; influence of family, friends and carers; seeking help; views and experiences of treatments; and how services might be improved. The topic guide (see Appendix 1) was modified as interviews progressed, through team discussions and in conjunction with our patient and public involvement (PPI) representatives, who also provided feedback upon recruitment materials. The terminology 'low' and 'worried' was used at the start of interviews to reduce any perceived stigma, but interviewers used the participant's own terminology as the interview progressed. We defined rather than simply named psychological therapies, as participants were often unsure about differences between these.

After each interview, demographic data and self-reported diagnoses of anxiety and/or depression were collected. Participants were provided with relevant local and national mental health service information, an Independent Age depression guide (Independent Age, 2017) and a £20 voucher as thanks. Concerns regarding suicidal ideation were discussed with the Co-PI and senior researcher (KW, an experienced academic GP) and communicated to the

For Peer Review Only

person's GP with their consent. Interviews were audio-recorded, with brief field notes, transcribed verbatim by an external company and anonymised and verified for accuracy by the interviewer (RF or PN).

We used thematic analysis to analyse our data from a constructivist perspective (Lincoln & Guba, 2003). Our team included two health services researchers (RF, KK), three academic GPs (KW, MB, PN), two psychologists (RG, SA) and two PPI members. All transcripts were read by RF and PN and at least one additional team member. RF developed a thematic framework, refined through team discussions and piloting by multiple team members. RF, PN and SA coded transcripts using NVivo 12 (QSR International Pty Ltd., 2018) according to the thematic framework in Appendix 2. Saturation was judged to have occurred after 26 interviews and two further interviews were carried out to confirm this. The study was approved by NHS Camden and Kings Cross Research Ethics Committee (ref 17/LO/1963).

Results

We recruited 28 participants with a mean age of 80.71 years (range 75-88) (see Table 1 for demographics). The majority of participants were female (n=19/28), White British (n=22/28), lived alone (n=17/28), from urban (n=14/28) and semi-rural locations (n=10/28), and owned their homes (n=16/28), but represented a range of marital statuses and educational levels. A depression diagnosis was self-reported by four participants, anxiety by four, and three reported having both. Seventeen had no self-reported diagnosis, although some of these reported taking antidepressants, and two were unsure if they had a diagnosis. The telephone screen had indicated nearly half experienced both anxiety and depressive symptoms, with the rest experiencing either anxiety or depressive symptoms, suggesting a range of mental health experiences were included.

For Peer Review Only

[Table 1 about here]

Participants reported a range of experiences associated with depression and anxiety. Many experienced transient low feelings that varied day-to-day according to their physical health, functioning and energy levels, and was often characterised by feeling ‘frustrated’ and ‘fed up’, underpinned by fears of worsening functioning. Others experienced more severe depression, including volatile emotions (e.g. ‘crying at the drop of a hat’) and feeling unable to cope. A small number had suicidal thoughts, but none reported specific plans (their GPs were contacted in two cases). Anxiety symptoms ranged from excessive worry over ‘silly little things’, such as sorting housework or people visiting, to strong fears about their future physical health, feeling constantly ‘on edge’ or specific anxieties (e.g. being unable to move). For a few people, unresolved anxieties about health also led to feeling low and depressed. Anxiety and depression were experienced as different constructs, but those with symptoms of both felt they interacted and rarely expressed preferences for different treatments for each. Separating preferences according to anxiety and depression was therefore difficult but is highlighted where possible throughout the results. Participants usually self-managed minor symptoms (to be reported in a separate paper), but six main themes emerged regarding their views about treatment: expectations of treatment, appropriateness of different treatments, promoting independence, connection, inclusivity of mental health services and endorsement of treatments.

Expectations of treatment

Participants generally had low expectations of their mood at this point in life and therefore the potential for treatment. Feeling low and worrying about health was normalised as part of declining physical health, and the ageing experience.

For Peer Review Only

> *[prospect of decline and death] all that's sort of ominous, you know. So, I'd be unrealistic if I was, if I was hilariously happy (83 White British M, no diagnosis).*

Expectations of the potential of treatments were based mainly upon past experiences of poor mental health and its treatment and the similarity of these to the present context. As earlier life anxiety narratives were typically dichotomised into nervous breakdowns (which might require institutionalisation) or normal, stressful situations (requiring no treatment), mild-moderate anxiety was normalised as a rational response to health problems and/or everyday issues (e.g. smashing a vase). This was consequently trivialised as 'silly', and participants made critical self-judgements about their anxiety, rather than perceiving it as a treatable medical condition:

> *Sometimes I feel so dreadful that I feel so anxious. And I think, "Oh, you are stupid," because I think I go up to the bedroom and want to do something moving and then I'll knock it all over (84 White British F, no diagnosis)*

For Peer Review Only

Nevertheless, anxiety could have a large impact on everyday functioning – some participants sat for hours worrying or crying, or woke in the night, whilst others stopped leaving the house alone for fear of falls, fainting, etc. A minority mentioned discussing anxiety in the context of health worries with their GP (more so if they also had depressive symptoms) but expectations of future treatment were lowered by the limited support available.

> *He's [GP] just told me not to worry. How can you stop worrying? They haven't got a tablet for it. (86 White British M, anxiety)*

Despite low expectations, those with stronger anxiety symptoms reported greater desperation and willingness to try a wider range of therapies to help reduce their symptoms:

> *Anything I can do [for the anxiety], I'll try and do to help. (76 Black Caribbean F, anxiety)*

Episodes of depression had a more marked presence throughout participants' life stories, attributed to trauma (e.g. sexual abuse, war memories, miscarriage), long term disability and bereavement. It had become part of some participants' identity and narratives, leading to resignation that it was simply something to live with at this point in life. As these participants had usually sought help before, depression was more readily perceived as a medical issue than anxiety, but treatment expectations were low:

> *I suppose because, because I've had all those issues from the very, for so many years, they're never going to go away, you know. (79 White British F, depression)*

Ongoing issues perpetuated by social factors beyond their control (e.g. caring, family conflict, finances) were a particular struggle for some. These were considered unamenable to treatment and led to feelings of hopelessness and desperation. This was compounded when recent help-seeking had resulted in only limited effects, with few reporting being offered further options.

> *I'm constantly up the GPs crying and carrying on. But they don't know how to help. How can [they] help, who can help? (86 WB F, anxiety and depression)*

For Peer Review Only

Some of these participants expressed suicidal ideas or a passive death wish - typically those with limited social support who had exhausted their coping skills.

An undercurrent within these discussions was an expectation that developing services further was unlikely in the current UK climate of austerity and an overstretched NHS, reinforced by reductions in local council services.

> *you can put forward any recommendation that you want, you can do all sorts of studies, as you most probably know, but your stumbling block is the authorities up there…nine times out of ten, it doesn't happen, does it? (80, White British M, depression and anxiety)*

Appropriateness of different treatments

Formal mental health services were only considered appropriate for severe symptoms. Those with mild symptoms expressed preferences for self-management, e.g. accessing social support through activity clubs and volunteering. The GP (regardless of relationship quality) was considered an appropriate first point of contact for moderate to severe mental health symptoms, as a provider of general health support and gatekeeper to other services:

> *He's my GP. And I would talk to him. (77 Black Caribbean F, no diagnosis)*

Treatment preferences varied considerably by individual, previous life experience and social situation. Some participants had clear preferences for psychological support (e.g. talking, problem solving), whilst many were unsure what was available:

> *I don't know. You know, I've never dealt with them people [psychologists]. (86 White British M, anxiety)*

For Peer Review Only

Counselling was construed as ‘talking to someone’ – potentially therapeutic for expressing emotions and gaining insight into feelings, but superfluous where participants had sufficient social support or understood why they felt this way.

> *You want empathy, you want a listening ear. Somebody who will listen. (80 Black Caribbean F, no diagnosis)*

> *I know why I’m feeling low. So, what is the counsellor going to tell me that I don’t already know. (87 Black Caribbean F, no diagnosis)*

Cognitive behavioural therapy (CBT) was not well understood and frequently confused with counselling. A couple had had positive experiences of CBT, but for others some CBT activities did not make sense (e.g. metaphors used). When CBT was described as improving coping skills through examining and reframing thought patterns, participants either felt their coping skills were sufficient or feared it might conflict with current coping mechanisms (mainly distraction) through reinforcing ‘sitting and thinking’, which they considered an important factor for worsening low and anxious feelings:

> *I think most people, the one thing you don’t want to do is think…if somebody is telling you another way to think through it, it’s another think process, isn’t it? (82 White British F, anxiety)*

Approximately two thirds of interviewees had taken antidepressants earlier in their lives, with some on long term prescriptions. Antidepressants were often considered incongruent with participants’ needs as they could not address the cause of their depression or anxiety and were occasionally associated with past suicide attempts. The assumption they would be offered antidepressants caused some to avoid sharing their low mood with their GP.

For Peer Review Only

Antidepressants were construed as making people ‘zombies’ and dulling emotions, necessary only when people were overwhelmed by their feelings:

> *[Prozac keeps me] Not on a good level, on a low level. But if it was taken away I’d be on no level, if you understand what I’m saying. (86 White British F, anxiety and depression)*

Promoting independence

Independence was a top priority for most participants. Any help-seeking could potentially threaten this; therefore therapies needed to facilitate independence and self-esteem. The strongly perceived link between antidepressants and dependency was viewed as particularly threatening, as well as meaning taking additional medication. Choosing not to use antidepressants, particularly when symptoms were less severe, was therefore seen by some as a sign of inner strength and control which could encourage self-esteem:

> *I wasn’t quite that bad, you know, I had the choice. (86 White British M, unsure re diagnosis)*

Talking therapies were perceived as less threatening to independence, in that they encouraged participants to reach their own solutions and supported their coping skills for depression and anxiety, in addition to, or instead of empathic listening.

> *I want somebody to listen and, and then advise me what you can do to help yourself and what you can’t do. (77 Black Caribbean F, no diagnosis)*

For some, practical advice and support to resolve daily hassles related to frailty and disability was particularly important, as these could profoundly impact upon people’s lives as ongoing

For Peer Review Only

stressors, particularly for anxiety. Being encouraged to take practical action was seen as more appropriate in this context, in order to both resolve the problem and improve self-esteem.

> *it doesn't help to talk about it, it helps to get it done. You know, I think talking about it just gives you more aggravation or more anxiety. (82 White British F, anxiety)*

Connection

Whilst seeking help for mental health through the GP did not depend upon a good relationship, support to treat both depression and anxiety was considered highly dependent upon the skills and personality of the person/therapist involved. This could greatly facilitate or obstruct engagement with treatment, and counsellors or social workers were often discussed in strong terms e.g. 'brilliant' or 'useless'.

> *social services came here, social worker. And she put it down to loneliness and I thought, if you had just that much of a clue what's gone on in my life, loneliness is not it (77 White British F, depression (unsure re. anxiety))*

What older people desired was for someone to listen well and provide an outside perspective, with genuine interest and the skills to facilitate older people to 'open up'.

> *I've always felt that when you're on the couch, you know, that the person, the counsellor has got the expertise to be able to delve and probe and perhaps bring forward things that you would shut away up here, which may or may not affect the way that you are now. (80 White British M, anxiety and depression)*

For Peer Review Only

This approach did not need to involve a particular profession, and a few named social workers and GPs as well as mental health professionals as potentially helpful. Occasionally other older people or adult children experiencing the onset of old age could provide support from a place of shared experience, although mental health was rarely explicitly referred to.

> *they do come and see you, which we do with each other, you know. They'll say, 'Thanks for the coffee, I feel better now.' (86 White British M, unsure diagnosis)*

Support groups could also have this effect:

> *just to meet people and to realise there are other people like you. It isn't just you that are having this 'Oh shit, like, you know, I can't do that anymore.' (82 WB F, anxiety)*

However, views about support groups varied according to individual preferences, and many felt formal group therapy might negatively affect their mood through fears of gossip or listening to others' problems:

> *There's enough of headcases running around you know to be sitting among them. (78 Irish M, no diagnosis)*

Face-to-face contact enhanced a sense of connection. Telephone appointments were precluded by hearing difficulties or fears of not knowing who they were speaking to. Video calling was seen as better as the person was visible, but this depended highly upon individual familiarity with technology and was still perceived as a hindrance to opening up.

For Peer Review Only

I think that both of those [telephones and video calling] give you far too many opportunities to hide behind things. (75 White British F, no diagnosis)

Inclusivity of mental health services

Methods of accessing services or information about mental health were often at odds with how older people preferred to access health services. Some participants with a strong interest in psychology were happy to access information and services online:

I've got a full thing on cognitive behavioural therapy there. I've got, I'm doing a course on it on the computer at the moment. (76 White British F, no diagnosis)

However, the vast majority of participants were limited in their use of technology and did not consider themselves very skilled, or did not own a computer. Many struggled to access practical services such as online shopping or banking, and felt that online self-referral to or delivery of mental health services would be similarly inaccessible.

one thing that really pisses me off is when people say, 'Oh yes, well just do it online.' No, no I don't do it online. (82 White British F, anxiety)

The preferred pathway was to seek help with mental health issues through their GP when symptoms became moderate-to-severe. In these cases, not wanting to bother the GP was a much less important barrier than being unable to access appointments (particularly with a familiar GP) or to find time within appointment(s) to prioritise discussion of mental health problems over physical ones.

For Peer Review Only

I was going to talk to the doctor that last time I went. And then, anyway it was, you know, I only had a ten-minute appointment and I thought no, I can't say it now because I don't want to say a lot. (79 White British F, depression)

Transport was a further consideration. Participants highlighted that any psychological services needed to be delivered at home, very locally or with transport provided. All the participants had at least some difficulty leaving their homes, and for some finances were a major factor. However, leaving the home was viewed as a top priority and therefore transport provision was sometimes preferred over home visits.

you [services] getting to them [older people] is fine, but I don't think it does them as much good as them getting to you. (82 White British F, anxiety)

Endorsement of treatments

Treatments needed to be endorsed by someone else to validate their appropriateness and increase participants' willingness to try them despite their lack of familiarity. Apart from the minority with an interest in psychology, most felt insufficiently knowledgeable to make treatment decisions.

I think that would be up to the doctor to decide you're bad enough and send you to somebody. (76 White British F, no diagnosis)

This endorsement rarely arose from any source apart from their GP for frail older people. It was rare that participants reported seeking mental health advice from books, magazines or the internet. Most acquired information through discussions with others with regard to general

For Peer Review Only

recommendations e.g. for gardeners or cleaners. However, although some reported sharing their problems, discussions around mental health support were rare and depended heavily on individual personalities and friendships. One participant described how she and two others in her social circle saw the same counsellor but never discussed this, and others expressed surprise when friends disclosed a depression diagnosis.

> *I didn't know she had it, she didn't tell anybody. She was just crying last week and she said she's got depression (82 White British F, anxiety)*

Whilst the third sector (voluntary organisations such as Age UK) was viewed as a positive resource for practical help, most people were unaware that some providers offered mental health support.

Discussion

We found frail older people with mild to severe symptoms of anxiety and/or depression had low expectations of their wellbeing at this point in life. Mild depressive symptoms and all severities of anxiety were normalised as part of multiple health issues and functional difficulties. Those with moderate to severe depression were open to seeking help, but those with anxiety were far more reluctant. Seeking treatment but not experiencing an improvement could lead to a marked sense of desperation. Treatments were perceived as appropriate when they clearly addressed the cause of someone's depression/anxiety, aligned with their current coping skills and focussed on facilitating independence and enhancing coping skills. The interpersonal relationship was particularly valued in all mental health support. The preferred method of accessing support (through their GP) often presented access barriers. Many frail older people wanted their treatment endorsed by their GP to legitimise this, as they felt lacking in mental health expertise.

URL: http:/mc.manuscriptcentral.com/camh
For Peer Review Only

Frail older people cite similar reasons for avoiding seeking mental health support as both the 'younger old' and adult populations, including stigma, normalising symptoms, threats to identity and failure to recognise a need for support (Doblyte & Jiménez-Mejías, 2017; Holm & Severinsson, 2014; Knight & Winterbotham, 2019; Mackenzie, Pagura, & Sareen, 2010). Whilst moderate to severe symptoms were a recognised cause for concern, mild depressive symptoms and anxiety were normalised and usually self-managed. This is likely to be appropriate where self-management strategies are working well, which in turn may help older people to maintain a sense of personhood and resist a 'frailty identity', which they associate with worse psychological health and social disengagement (Warmoth et al., 2016). However, low expectations and being resigned to living with health needs they think cannot be met are reported for a range of age-related issues (Walters, Iliffe, & Orrell, 2001). Whilst a previous US survey showed that those aged 85+ preferred supportive therapy for anxiety rather than CBT (Mohlman, 2011), participants in our study also expressed a desire for coping strategies, practical advice and suggestions in addition to empathic listening. 'Being heard' and aligning interventions with older people's needs were also highlighted as important components by older people with multimorbidity (Bayliss, Edwards, Steiner, & Main, 2008).

Strengths and Weaknesses

We captured a wide range of views from frail older people, who varied according to socioeconomic status, ethnicity, degree of frailty and symptom severity. In contrast to previous studies focussing mainly on depression, we placed equal emphasis on depression and anxiety. Anxiety in frail older adults has largely been neglected in comparison to depression and this is the first study to the authors' knowledge to qualitatively explore help-seeking and treatment preferences for anxiety in frail older people. Our topic guides and themes were developed in conjunction with our PPI representatives and all team members

For Peer Review Only

contributed to the interpretation, offering a range of perspectives. We approached the research from a constructivist perspective (Lincoln & Guba, 2003), assuming depression and anxiety and its treatments would be conceptualised and experienced in a variety of ways.

However, we could only provide limited insight into possible therapeutic approaches, as participants found it difficult to hypothesise and articulate what might be helpful in any detail, even when specific therapies were described or they had experienced them previously. We did not collect specific data on treatments currently or previously experienced. Although we tried to sample people with more severe frailty experiencing formal home care or residential care support, we had a low response from this population, which may reflect the greater cognitive impairment commonly found. We sampled a variety of ethnicities, but did not recruit anyone who could not speak English. As less than a third of our sample self-reported a formal mental health diagnosis, the experiences of those with more severe symptoms may not have been fully explored. We were unable to access participants' medical records to verify self-reported diagnosis, but this may be under-reported as two thirds of our sample discussed taking antidepressants at some point in their life.

Implications for Research and Practice

In terms of practice, our study suggests that older people expect mental health care recommendations to occur within the context of primary care, and that improving access to primary care may be helpful in encouraging them to raise mental health concerns and try treatments offered, despite some pessimism as to whether they might work. However, GPs and other healthcare professionals commonly cite difficulties in addressing the complexities of late-life depression in short appointments, particularly when complex physical health issues are present (Rachael Frost et al., 2019). In this way, ensuring consistent pro-active

For Peer Review Only

screening for both depression and anxiety as part of complex care or frailty reviews is likely to be helpful.

Both health and social care providers need to maintain an awareness of anxiety as well as depression. Management of anxiety in frail older people has frequently been neglected in research, despite evidence suggesting comorbid anxiety impacts negatively upon treatment outcomes in late-life depression (Tunvirachaisakul et al., 2017). Participants in our study were much less likely to seek support or medical advice for anxiety symptoms, which reflects the difficulty recognising anxiety and the reluctance to discuss it found in other qualitative studies of older people with anxiety (Kingstone et al., 2017; Knight & Winterbotham, 2019). It may be that there is less public discourse relating to anxiety than depression which older people can use to explain and legitimise their feelings, although a few were comfortable describing their symptoms as 'anxiety'. Evidence suggests older people may use language such as 'fret' or 'concern' rather than 'worry' or 'anxiety' to describe symptoms (Stanley & Novy, 2000) and want to appear resilient. Awareness of late life anxiety as a treatable condition needs to be raised in frailer older populations.

Currently, psychological therapy access rates for older people are lower than expected, particularly for those aged 75+ (NAPT, 2013). This may relate to access barriers as highlighted in our work, and facilitating access through increased GP referrals may overcome this to some extent. Technological solutions such as telephone or video call support do not seem widely accepted at present in this and other studies (Moult, Burroughs, Kingstone, & Chew-Graham, 2018), although this appears to be changing as newer generations of older people use both healthcare professionals and the internet as sources of information regarding anxiety (Zapata et al., 2018). With regards to social prescribing, older people preferred to use their own resources to identify shared interest groups and were less keen on (mental) health

For Peer Review Only

support groups. Potential treatments also need to concord with older people's own coping mechanisms (e.g. acceptance, distraction), which therapies such as CBT could conflict with. Action research of psychological therapies targeted at the 'younger old' suggests that CBT homework was viewed as unhelpful and rarely carried out, whilst personalised information is preferred over abstract concepts (Richardson & Reid, 2006). Although CBT is effective for depression and anxiety in non-frail older adults when compared to treatment-as-usual or waiting list control conditions (Gould et al., 2012a, 2012b), effect sizes are typically smaller than for those who are younger and they have rarely been investigated in frailer populations.

Physical health played an important role in relation to mood, and our work suggests that therapies which encourage ways of coping with physical health problems and promoting independence may be more acceptable and relevant to this population. Problem-Solving Therapy (PST; a structured approach to problem-solving, aiming to both improve self-esteem to resolve issues and to reduce the causes or exacerbators of depressive symptoms (Mynors-Wallis, 2005)), may be helpful and has some evidence of effectiveness for depression in frail older people (R Frost et al., 2018). Other approaches such as Acceptance and Commitment Therapy (ACT) which focuses on increasing acceptance of challenging experiences rather than trying to change or remove them, as well as increasing function, may also be relevant (Hayes, Strosahl, & Wilson, 1999). Older people with chronic pain are more clinically responsive to ACT than CBT (Wetherell et al., 2015), and pilot studies suggest it is acceptable to older people with anxiety (Wetherell et al., 2011). Evaluations of these promising therapies for frail older people with depression/anxiety are therefore needed in a UK NHS setting, particularly those that focus on addressing anxiety.

For Peer Review Only

Conclusion

Our interviews with frail older people with anxiety and/or depressive symptoms suggested that frail older people tend to seek medical help for more severe symptoms, but find it difficult to know when to seek help for symptoms of anxiety, so awareness of potential treatments for anxiety needs to be promoted in later life. Expectations of mental health treatments and wellbeing in later life were low, but older people felt they often had insufficient knowledge to judge this and so endorsement from a GP could facilitate uptake of therapies. Good support was judged to be provided face to face, by persons who could connect with them through their shared experience, and provide empathic listening to encourage opening up, regardless of their mental health training. Such therapy would have to empower rather than problematize mental health issues by helping them maintain a sense of personhood, independence and ability to cope with physical health issues. Further evaluation is needed of novel therapies that may be more applicable for the experiences of this population (e.g. ACT, PST).

Disclosure statement

The authors declare no conflict of interest.

Data availability statement

This is a qualitative study and therefore the data generated is not suitable for sharing beyond that contained within the report. Further information can be obtained from the corresponding author.

For Peer Review Only

References

Bayliss, E. A., Edwards, A. E., Steiner, J. F., & Main, D. S. (2008). Processes of care desired by elderly patients with multimorbidities. *Family Practice*, *25*(4), 287–293.

Benraad, C. E. M., Kamerman-Celie, F., van Munster, B. C., Oude Voshaar, R. C., Spijker, J., & Olde Rikkert, M. G. M. (2016). Geriatric characteristics in randomised controlled trials on antidepressant drugs for older adults: a systematic review. *International Journal of Geriatric Psychiatry*, *31*(9), 990–1003.

Braam, A. W., Copeland, J. R. M., Delespaul, P. A. E. G., Beekman, A. T. F., Como, A., Dewey, M., … Skoog, I. (2014). Depression, subthreshold depression and comorbid anxiety symptoms in older Europeans: Results from the EURODEP concerted action. *Journal of Affective Disorders*, *155*(1), 266–272.

Clegg, A., & Hassan-Smith, Z. (2018). Frailty and the endocrine system. *The Lancet Diabetes & Endocrinology*, *6*(9), 743–752. https://doi.org/10.1016/S2213-8587(18)30110-4

Clegg, A., Young, J., Iliffe, S., Rikkert, M. O., & Rockwood, K. (2013). Frailty in elderly people. *The Lancet*, *381*(9868), 752–762.

Collard, R. M., Boter, H., Schoevers, R. A., & Oude Voshaar, R. C. (2012). Prevalence of frailty in community-dwelling older persons: A systematic review. *Journal of the American Geriatrics Society*, *60*(8), 1487–1492.

Corcoran, J., Brown, E., Davis, M., Pineda, M., Kadolph, J., & Bell, H. (2013). Depression in older adults: a meta-synthesis. *Journal of Gerontological Social Work*, *56*(6), 509–534. https://doi.org/10.1080/01634372.2013.811144

For Peer Review Only

Dent, E., & Hoogendijk, E. (2014). Psychosocial factors modify the association of frailty with adverse outcomes: a prospective study of hospitalised older people. *BMC Geriatrics*, *14*(1), 108. Retrieved from http://www.biomedcentral.com/1471-2318/14/108

Djernes, J. K., Gulmann, N. C., Foldager, L., Olesen, F., & Munk-Jørgensen, P. (2011). 13 Year Follow Up of Morbidity, Mortality and Use of Health Services Among Elderly Depressed Patients and General Elderly Populations. *The Australian and New Zealand Journal of Psychiatry*, *45*(8), 654–662.

Doblyte, S., & Jiménez-Mejías, E. (2017). Understanding Help-Seeking Behavior in Depression: A Qualitative Synthesis of Patients' Experiences. *Qualitative Health Research*, *27*(1), 100–113.

Fried, L. P., Tangen, C. M., Walston, J., Newman, A. B., Hirsch, C., Gottdiener, J., … McBurnie, M. A. (2001). Frailty in older adults: Evidence for a phenotype. *Journals of Gerontology: Medical Sciences*, *56A*(3), M146–M156.

Frost, R., Bauernfreund, Y., & Walters, K. (2018). Non-pharmacological interventions for depression/anxiety in older adults with physical comorbidities affecting functioning: systematic review and meta-analysis. *International Psychogeriatrics*. https://doi.org/doi:10.1017/S1041610218001564

Frost, R., Beattie, A., Bhanu, C., Walters, K., & Ben-Shlomo, Y. (2019). Management of depression and referral of older people to psychological therapies: a systematic review of qualitative studies. *British Journal of General Practice*, *69*(680), e171–e181. https://doi.org/10.3399/bjgp19X701297

For Peer Review Only

Gould, R., Coulson, M., & Howard, R. (2012a). Cognitive behavioral therapy for depression in older people: A meta-analysis and meta-regression of randomized controlled trials. *Journal of the American Geriatrics Society*, *60*(10), 1817–1830.

Gould, R., Coulson, M., & Howard, R. (2012b). Efficacy of cognitive behavioral therapy for anxiety disorders in older people: A meta-analysis and meta-regression of randomized controlled trials. *Journal of the American Geriatrics Society*, *60*(2), 218–229.

Gum, A., Areán, P., Hunkeler, E., Tang, L., Katon, W., Hitchcock, P., … Unützer, J. (2006). Depression treatment preferences in older primary care patients. *The Gerontologist*, *46*(1), 14–22.

Gutiérrez-valencia, C. M., Hospitalario, C., & Irunlarrea, D. N. C. (2018). *SYSTEMATIC REVIEW AND META – ANALYSIS The relationship between frailty and polypharmacy in older people : A systematic review*. https://doi.org/10.1111/bcp.13590

Hayes, S. C., Strosahl, K. D., & Wilson, K. G. (1999). *Acceptance and Commitment Therapy: An experiential approach to behavior change.* New York: The Guilford Press.

Holm, A. L., & Severinsson, E. (2014). Surviving depressive ill-health: A qualitative systematic review of older persons' narratives. *Nursing and Health Sciences*, *16*(1), 131–140. https://doi.org/10.1111/nhs.12071

Independent Age. (2017). Dealing with depression: How to recognise the signs and the action you can take. Retrieved September 3, 2018, from https://www.independentage.org/information/advice-guides-factsheets-leaflets/dealing-depression

Kingstone, T., Burroughs, H., Bartlam, B., Ray, M., Proctor, J., Shepherd, T., … Chew-

For Peer Review Only

Graham, C. A. (2017). Developing a community-based psycho-social intervention with older people and third sector workers for anxiety and depression: A qualitative study. *BMC Family Practice*, *18*(1), 1–9. https://doi.org/10.1186/s12875-017-0648-7

Kirkham, J. G., Choi, N., & Seitz, D. P. (2016). Meta-analysis of problem solving therapy for the treatment of major depressive disorder in older adults. *International Journal of Geriatric Psychiatry*, *31*(5), 526–535.

Knight, B. G., & Winterbotham, S. (2019). Rural and urban older adults' perceptions of mental health services accessibility. *Aging & Mental Health*, *0*(0), 1–7. https://doi.org/10.1080/13607863.2019.1576159

Kok, R. M., Nolen, W. A., & Heeren, T. J. (2012). Efficacy of treatment in older depressed patients: A systematic review and meta-analysis of double-blind randomized controlled trials with antidepressants. *Journal of Affective Disorders*, *141*(2–3), 103–115. https://doi.org/10.1016/j.jad.2012.02.036

Lan, X., Xiao, H., & Chen, Y. (2017). Effects of life review interventions on psychosocial outcomes among older adults: A systematic review and meta-analysis. *Geriatrics & Gerontology International*, *17*, 1344–1357. Retrieved from http://doi.wiley.com/10.1111/ggi.12947

Landreville, P., Landry, J., Baillargeon, L., Guérette, A., & Matteau, É. (2001). *Older Adults ' Acceptance of Psychological and Pharmacological Treatments for Depression*. *56*(5), 285–291.

Li, C., Friedman, B., Conwell, Y., & Fiscella, K. (2007). Validity of the Patient Health Questionnaire 2 (PHQ-2) in identifying major depression in older people. *Journal of the*

For Peer Review Only

American Geriatrics Society, *55*(4), 596–602.

Lincoln, Y., & Guba, E. (2003). Paradigmatic Controversies, Contradictions and Emerging Confluences. In N. Denzin & Y. Lincoln (Eds.), *The Landscape of Qualitative Research: Theories and Issues* (2nd Ed, pp. 253–291). London: SAGE Publications Inc.

Mackenzie, C. S., Pagura, J., & Sareen, J. (2010). Correlates of perceived need for and use of mental health services by older adults in the collaborative psychiatric epidemiology surveys. *The American Journal of Geriatric Psychiatry*, *18*, 1103–1115.

Meeks, T. W., Vahia, I. V, Lavretsky, H., Kulkarni, G., & Jeste, D. V. (2011). A tune in "a minor" can "b major": A review of epidemiology, illness course, and public health implications of subthreshold depression in older adults. *Journal of Affective Disorders*, *129*(1–3), 126–142. Retrieved from https://search.proquest.com/docview/855900130?accountid=14511

Mitchell, A. J., Rao, S., & Vaze, A. (2010). Do primary care physicians have particular difficulty identifying late-life depression? A meta-analysis stratified by age. *Psychotherapy and Psychosomatics*, *79*(5), 285–294. https://doi.org/10.1159/000318295

Mohlman, J. (2011). A community based survey of older adults' preferences for treatment of anxiety. *Psychology and Aging*, *27*(4), 1182–1190.

Moult, A., Burroughs, H., Kingstone, T., & Chew-Graham, C. A. (2018). How older adults self-manage distress - Does the internet have a role? A qualitative study. *BMC Family Practice*, *19*(1), 1–8. https://doi.org/10.1186/s12875-018-0874-7

Mynors-Wallis, L. (2005). *Problem-solving treatment for anxiety and depression: A practical guide*. Oxford: Oxford University Press.

For Peer Review Only

NAPT. (2013). *Second Round of the National Audit of Psychological Therapies for Anxiety and Depression (NAPT) National Report November 2013*. (November), 169. Retrieved from http://www.rcpsych.ac.uk/pdf/NAPT second round National report website 28-11-13v3.pdf#page=4&zoom=auto,-82,390

Ni Mhaolain, A. M., Fan, C. W., Romero-Ortuno, R., Cogan, L., Cunningham, C., Kenny, R. A., & Lawlor, B. (2012). Frailty, depression, and anxiety in later life. *Int Psychogeriatr*, *24*(8), 1265–1274.

Potter, G. G., McQuoid, D. R., Whitson, H. E., & Steffens, D. C. (2016). Physical frailty in late-life depression is associated with deficits in speed-dependent executive functions. *International Journal of Geriatric Psychiatry*, *31*(5), 466–474.

QSR International Pty Ltd. (2018). *NVivo qualitative data analysis software. Version 12.* QSR International Pty Ltd.

Richardson, L., & Reid, C. (2006). “I’ve lost my husband, my house and I need a new knee ... why should I smile?”: Action research evaluation of a group cognitive behavioural therapy program for older adults with depression. *Clinical Psychologist*, *10*(2), 60–66.

Shaw, R. L., Gwyther, H., Holland, C., Bujnowska, M., Kurpas, D., Cano, A., … Avanzo, B. D. (2018). Understanding frailty: meanings and beliefs about screening and prevention across key stakeholder groups in Europe. *Ageing & Society*, *38*, 1223–1252.

Stanley, M. A., & Novy, D. M. (2000). Cognitive-behavior therapy for generalized anxiety in late life: An evaluative overview. *Journal of Anxiety Disorders*, *14*(2), 191–207.

Tunvirachaisakul, C., Gould, R. L., Coulson, M. C., Ward, E. V., Reynolds, G., Gathercole, R. L., … Howard, R. J. (2017). Predictors of treatment outcome in depression in later

For Peer Review Only

life: A systematic review and meta-analysis. *Journal of Affective Disorders*, *227*(April), 164–182.

Vaughan, L., Corbin, A. L., & Goveas, J. S. (2015). Depression and frailty in later life: A systematic review. *Clinical Interventions in Aging*, *10*, 1947–1958.

Walters, K., Falcaro, M., Freemantle, N., King, M., & Ben-Shlomo, Y. (2018). Sociodemographic inequalities in the management of depression in adults aged 55 and over: an analysis of English primary care data. *Psychological Medicine*, *48*(9), 1504–1513.

Walters, K., Iliffe, S., & Orrell, M. (2001). *An exploration of help-seeking behaviour in older people with unmet needs*. *18*(3), 277–282.

Warmoth, K., Lang, I., Phoenix, C., Abraham, C., Andrew, M., Hubbard, R., & Tarrant, M. (2016). 'Thinking you're old and frail': a qualitative study of frailty in older adults. *Ageing and Society*, *36*, 1483–1500.

Wetherell, J. L., Petkus, A. J., Alonso-Fernandez, M., Bower, E., Steiner, A., & N, A. (2015). Age moderates response to acceptance and commitment therapy vs. cognitive behavioral therapy for chronic pain. *International Journal of Geriatric Psychiatry*, *31*(3), 302–308.

Wild, B., Eckl, A., Herzog, W., Niehoff, D., Lechner, S., Maatouk, I., … Loewe, B. (2014). Assessing generalized anxiety disorder in elderly people using the GAD-7 and GAD-2 scales: results of a validation study. *The American Journal of Geriatric Psychiatry*, *22*(10), 1029–1038.

Wolitzky-Taylor, K., Castriotta, N., Lenze, E., Stanley, M., & Craske, M. (2010). Anxiety disorders in older adults: A comprehensive review. *Depression and Anxiety*, *27*(2), 190–

For Peer Review Only

211.

Zapata, A. M. L., Beaudreau, S. A., O’Hara, R., Bereknyei Merrell, S., Bruce, J., Garrison-Diehn, C., & Gould, C. E. (2018). Information-Seeking about Anxiety and Perceptions about Technology to Teach Coping Skills in Older Veterans. *Clinical Gerontologist*, *41*(4), 346–356. https://doi.org/10.1080/07317115.2017.1359716

For Peer Review Only

1 **Table 1 Demographic details of participants**

Demographics	Number	
**Age mean (SD)**<br>**Range**	80.71 (4.07) years<br>75-88 years	
**Gender (male:female)**	9:19	
**Location**	Semi-rural	10
	Surburban	4
	Urban	14
**Born in**	UK	22
	Trinidad	2
	Mauritius	1
	Ireland	1
	Jamaica	2
	Unknown	1
**Ethnicity**	White British	22
	Black Caribbean	4
	White Irish	1
	Indian	1
**Living situation**	Alone	17
	With spouse	8
	With other family	3
**Marital status**	Widowed	10
	Married	8
	Separated	2
	Divorced	7
	Single	1
**Current housing**	Owner-occupier	16
	Council rented	7
	Housing association rented	2
	Sheltered housing	2
	Private rented	1
**Age completed**	Before the age of 15 yrs	8

For Peer Review Only

**education**	Aged 15-16 yrs	7
	Aged 17-20 yrs	6
	Aged over 21 yrs	7
**Type of pension**	State	28
	Employer	17
	Private	3
	Pension credits	10
**Telephone screen**	PHQ-9 positive screen only	10
	GAD-2 positive screen only	5
	Both	13
**Diagnosis**	Depression only	4
	Depression (unsure re anxiety)	1
	Anxiety only	4
	Both	3
	Neither	16
	Unsure	1

1

For Peer Review Only

Mood in Later Life: Topic Guide

Thank you for taking part in this study. We know that feeling or low or stressed can be common in later life, particularly when people find everyday activities more of a struggle, and this can lead to a lower quality of life and further physical difficulties. We would like to understand your experiences of mood and wellbeing in later life so we can develop better ways of providing support to people in future through the NHS or social care. If there are any questions you would prefer not to answer, please let me know and we can skip over it.

Mental health experiences in later life

- What sort of things do you do in a **normal week**? (e.g. seeing friends, going for a walk)
- How are you **feeling in yourself** at the moment?
- Have you felt low **recently**? Can you tell me more about this?
- Have you felt anxious recently? Can you tell me more about this?
- **How often** do you feel this way?
- Have you been through periods where you have felt low or anxious earlier in your life [under 65]? Could you tell me about that?
- [If yes] How does that compare to how you feel now?

Self-managing feelings of stress or depression [depending on main issue]

- **When you feel low [worried], what do you do**?
- What do you think is the **most helpful** way to deal with feeling low [anxious]? What about the least helpful?
- Is this the same as when you feel anxious [low]?
- How do your **current health problems** affect your mood?
- Have you looked for any information regarding ways to cope when you felt low/worried?
  - If yes, what sources of information did you seek? (prompt for accessing information online)
  - If no, why not?
- [If applicable] How does your previous experience of feeling low or anxious affect how you deal with anxiety/feeling low now?
- How well do your previous coping mechanisms help you now? Are there ways of coping that you've used previously that you can no longer use now?
  - Prompts: Exercise? Socialising with others?
- Is feeling low or worried something you ever talk about with others?
  - If so, who?
- What effect do **family and friends** have upon your mood?
  - Explore intergenerational linkages and effects of extended family
  - Explore effects of loneliness and how management differs
- (If receiving care) what effect do your **carers** have on your mood?
- What effects do your activities/beliefs [specify from earlier conversation] have upon your mood?
  - Explore effects of religion (meaning and community/support)
  - Explore meaning of any projects/activities
- What effects do you current situation have on your mood?
  - Explore effects of finances (caring?)
- **In an ideal world, what would help you to cope better with your worries/low mood?**

For Peer Review Only

Mood in Later Life: Topic Guide

- **What do you want out of life at this point in life?**
    - What do you think is holding you back from this?
    - What might help address this?
    - How could you imagine someone else being involved?

Support from NHS services

- Have you **spoken to anyone** e.g. your GP about feeling low/worried? What happened?
- If yes: What prompted you to seek support for your mood?
- If no: Are there any particular reasons why you haven't spoken to anyone about this?
- If threshold: explore what does (e.g. 'being desperate', 'black hole') that mean to you?

General: Sometimes a GP or nurse can refer you on to another person to help support you with your mood e.g. a wellbeing service, counsellor or psychologist, a psychiatrist, social activities. Have you ever been referred to one of these services?

- How did you feel about being offered this service? [prompts: concerns, expectations, prepared to try it?]
- What were your experiences of this service?
- What was helpful? Unhelpful? [prompts: ease of use, relationship with therapist, setting, side effects, how did this affect your experiences?]

If need further prompting re **specific services**:

- Sometimes your GP will prescribe you tablets such as antidepressants when you are feeling low. What are your views about these? [prompt: ease of use, side effects]
- Sometimes your doctor [or social worker?] can refer you to a service where you can meet a group of people and do an activity e.g. gardening, or a befriending service. This is called social prescribing. What do you think about this? [prompt: appropriate for needs?]
- Some people can also be referred to talking therapies, where someone will ask them about their mood and help them develop ways to overcome feelings of stress or low mood in the future. What do you think about this sort of therapy? [prompt: appropriateness, setting, therapist]

[*Prompts*: opinions, experiences of, how did this help (or not), would you use again]

Services could include:

- **Phone support**
- **Support groups**
- **Support from peers**
- **Skype support**
- **Counselling**
- **CBT (coping skills for feeling low/when you can't stop worrying)**
- **PST (solving everyday problems such as X that may be getting you down)**
- **Antidepressants**
- **Social groups**

URL: http:/mc.manuscriptcentral.com/camh
For Peer Review Only

Mood in Later Life: Topic Guide

A new service

- **Would you be willing to try anything (else) to help with your mood?**
- **Who** would you feel most comfortable talking to if you were feeling depressed?
    - Prompt: healthcare professional, family, friend, care worker
    - Specific prompts: GP, social worker, carer, nurse, friend, family member, family carer, occupational therapist, counsellor, psychologist, psychiatrist
    - Mental health specialist vs non-specialist person
- **Where** would be a good place to talk to someone?
- **How often** would you want to see them?
- What do you think about someone talking to you about your mood over the phone?
- What do you think about using online services to help cope with mood? e.g. a video conversation, skype or email, or reading about how people cope with your situation?
    - What would encourage you to use these online services? (e.g. someone by your side to demo how to operate, user-friendly interface)
    - If not, why not?
- If we developed a new service, should it be focussed only on mood or on **other things** too? Like what? [prompts: socialising, activities]
- What would make you **more likely to use** a service like this? Less likely?
- Would it be helpful for anyone else to be involved e.g. friend, relative? In what way?
- How could we ensure people would use this service?

Other prompts

- Do you know **anyone else who has felt this way**? How does it affect them? What do you think would help?

Thank you for taking part in this study. We will add your interview to others we've already collected, which will help us improve and develop services for low mood and anxiety in future.

Voucher + list of services

For Peer Review Only

Thematic Framework Final 30.5.18

Thematic Framework

Current experiences of anxiety

- Day-to-day worries
- Worries about the future and death
- Worries about health
- Feelings about feeling anxious
- Previous experiences of anxiety
- Others' experiences of anxiety
- Perceptions of causes
- Impact of anxiety

Current experiences of depression

- Emotions
- Thoughts about the future (including suicidal thoughts)
- Lack of motivation (particularly to go out)
- Frustration
- Feelings about feeling depressed
- Previous experiences of depression
- Others' experiences of depression
- Perceptions of causes
- Impact of depression

Influencing contextual factors

- Bereavement
- Family support (including problems/dependency on family)
- Insomnia/Sleep issues
- Finances
- Non-family support network (including isolation and loneliness)
- Environment (e.g. neighbours, local area)
- Role changes and identity
- Living situation/marriage and caring responsibilities
- Attitude towards ageing and feeling part of a shared 'older person' experience (inc others' experiences of ageing and 'not feeling old')
- Attitude towards society and younger generations (sense of belonging in time?)
- Sense of belonging in culture (may relate to above?)
- Addressing basic and instrumental needs
- Hobbies, activities and going out
- Sitting and thinking too much
- Volunteering and altruism
- Future expectations, goals and priorities
- Importance of appearance

Impact of physical health

- Falls and fear of falling
- Threat of new diagnoses (e.g. cancer scare)

For Peer Review Only

Thematic Framework Final 30.5.18

- Health fears and expectations (including others' experience of ageing)
- Recent health events (e.g. MI, stroke)
- Ongoing conditions and their impact
- Cognitive conditions
- Medications
- Disability/functional impairment
- Pain
- Attitudes/response to physical health experiences (acceptance, frustration etc)

Life history

- Early and mid-life loss and bereavement (partner, adult children, friends, miscarriage)
- War experiences
- Early and mid-life experience of another mental illness
- Early childhood and family
- Marriage and own family
- Personality, values and priorities
- Previous roles in life (work, caring for family)
- Previous good life experiences
- Religion and beliefs
- Early and mid-life experiences of physical ill health

Self-managing low mood and anxiety

- Being creative (writing, drawing, photography)
- Embracing emotions (wallow for a while, wait for it to pass, have a 'good cry')
- Problem solve/deal with everyday issues
- Activities and distraction (including eating, going out, chores, reading, TV, exercise)
- Social activities – shared interest and purely social
- Avoidance of activity (inc talking about other things, not going out in response to fear, asking another person to do an activity)
- Talking about it (to: friends, family, providers (carers, sheltered housing wardens etc), those with shared experiences, deceased partner)
- Humour
- Philosophising and acceptance (including making sense of things, downward comparisons, living for today)
- Following a routine (more preventative?)
- Holidays and respite
- Meditation/breathing/visualisation/good memories
- Religion (prayer, trusting in God, shouting at God, seeking support from church community)
- Pets
- Alcohol
- External prompts (appointments, friends)
- Previously helpful strategies that they can no longer use
- Dietary supplements etc

For Peer Review Only

Thematic Framework Final 30.5.18

Reasons for self-managing mental health

- Maintain independence
- Feel able to self-manage sufficiently
- Sufficient support network
- Professional support cannot help
- No one available to help
- Stigma
- Fear of consequences (e.g. being put in care home, prescribed antidepressants)
- Personhood/image (not the person want to present to the world)
- Healthcare system too difficult to navigate
- Previous treatment failed

Finding information (including but not exclusive to mental health)

- Internet, tablets and smartphones (more about staying in touch?)
- Family and friends
- Practical things (e.g. cleaner) vs mental health
- GP
- TV programmes
- Books, newspapers and magazines
- Courses
- How would like to find out information

Help seeking in primary care

- Attitude to talk to GP about mental health
- Detected by GP (in a consultation for something else)
- Life events (loss/bereavement/spouse move to care home – may overlap with above)
- Severity/need
- Relationship with GP
- Not wanting to bother GP
- Fear of antidepressant prescription
- GP ability to do something about it
- Access barriers (time, continuity, gatekeeping)

Attitudes to mental health treatment (inc others' experiences)

- General views around treatment
- General views around talking to someone (when no specific provider mentioned)
- Willingness to try (including vs nothing can help)
- Perceptions of psychiatry
- Perceptions of group talking therapies
- Perceptions of individual talking therapies
- Perceptions of antidepressants

Experiences of mental health treatments

- Decision-making (passive vs active role)
- Psychiatry
- Experiences of group talking therapies

For Peer Review Only

Thematic Framework Final 30.5.18

- Experiences of individual talking therapies (code counselling vs CBT if possible)
- Previous experience of medication for mood

Other health care experiences

- Experience of physical health treatments
- Insomnia treatments
- “the system” – health (fairness, waiting times, lack of funds, reciprocity etc)
- Ageism in healthcare
- Support from long term condition providers that impacts on mental health (e.g. admiral nurses providing point of contact)

Community services

- Perceptions of social prescribing referrals from healthcare professionals
- “the system” – local authority and access
- Paid support (inc carers, cleaners etc)
- Support within sheltered housing environments
- Support groups (mental and physical health)
- Shared interest groups
- Social groups
- Other support services (e.g. Age UK)
- Others’ experiences of community services
- Transport

New service characteristics (including hypothetical and past experiences)

- Helpful components
- Unhelpful components
- Tailoring to person or situation
- Healthcare professional characteristics
- Use of technology for delivery
- One-to-one vs group
- Timing, duration and continuity
- How to access

For Peer Review Only
