# Supplementary material for: Supporting frail older people with depression and anxiety: a qualitative study
Source: Aging Ment Health. 2019 Oct 16;24(12):1977–84. doi: 10.1080/13607863.2019.1647132 (PMC8842711; doi:10.1080/13607863.2019.1647132)
Supplement: Supplemental Material [file CAMH_A_1647132_SM8096.docx]

**Thematic Framework**

Current experiences of anxiety

- Day-to-day worries
- Worries about the future and death
- Worries about health
- Feelings about feeling anxious
- Previous experiences of anxiety
- Others’ experiences of anxiety
- Perceptions of causes
- Impact of anxiety

Current experiences of depression

- Emotions
- Thoughts about the future (including suicidal thoughts)
- Lack of motivation (particularly to go out)
- Frustration
- Feelings about feeling depressed
- Previous experiences of depression
- Others’ experiences of depression
- Perceptions of causes
- Impact of depression

Influencing contextual factors

- Bereavement
- Family support (including problems/dependency on family)
- Insomnia/Sleep issues
- Finances
- Non-family support network (including isolation and loneliness)
- Environment (e.g. neighbours, local area)
- Role changes and identity
- Living situation/marriage and caring responsibilities
- Attitude towards ageing and feeling part of a shared ‘older person’ experience (inc others’ experiences of ageing and ‘not feeling old’)
- Attitude towards society and younger generations (sense of belonging in time?)
- Sense of belonging in culture (may relate to above?)
- Addressing basic and instrumental needs
- Hobbies, activities and going out
- Sitting and thinking too much
- Volunteering and altruism
- Future expectations, goals and priorities
- Importance of appearance

Impact of physical health

- Falls and fear of falling
- Threat of new diagnoses (e.g. cancer scare)
- Health fears and expectations (including others’ experience of ageing)
- Recent health events (e.g. MI, stroke)
- Ongoing conditions and their impact
- Cognitive conditions
- Medications
- Disability/functional impairment
- Pain
- Attitudes/response to physical health experiences (acceptance, frustration etc)

Life history

- Early and mid-life loss and bereavement (partner, adult children, friends, miscarriage)
- War experiences
- Early and mid-life experience of another mental illness
- Early childhood and family
- Marriage and own family
- Personality, values and priorities
- Previous roles in life (work, caring for family)
- Previous good life experiences
- Religion and beliefs
- Early and mid-life experiences of physical ill health

Self-managing low mood and anxiety

- Being creative (writing, drawing, photography)
- Embracing emotions (wallow for a while, wait for it to pass, have a ‘good cry’)
- Problem solve/deal with everyday issues
- Activities and distraction (including eating, going out, chores, reading, TV, exercise)
- Social activities – shared interest and purely social
- Avoidance of activity (inc talking about other things, not going out in response to fear, asking another person to do an activity)
- Talking about it (to: friends, family, providers (carers, sheltered housing wardens etc), those with shared experiences, deceased partner)
- Humour
- Philosophising and acceptance (including making sense of things, downward comparisons, living for today)
- Following a routine (more preventative?)
- Holidays and respite
- Meditation/breathing/visualisation/good memories
- Religion (prayer, trusting in God, shouting at God, seeking support from church community)
- Pets
- Alcohol
- External prompts (appointments, friends)
- Previously helpful strategies that they can no longer use
- Dietary supplements etc

Reasons for self-managing mental health

- Maintain independence
- Feel able to self-manage sufficiently
- Sufficient support network
- Professional support cannot help
- No one available to help
- Stigma
- Fear of consequences (e.g. being put in care home, prescribed antidepressants)
- Personhood/image (not the person want to present to the world)
- Healthcare system too difficult to navigate
- Previous treatment failed

Finding information (including but not exclusive to mental health)

- Internet, tablets and smartphones (more about staying in touch?)
- Family and friends
- Practical things (e.g. cleaner) vs mental health
- GP
- TV programmes
- Books, newspapers and magazines
- Courses
- How would like to find out information

Help seeking in primary care

- Attitude to talk to GP about mental health
- Detected by GP (in a consultation for something else)
- Life events (loss/bereavement/spouse move to care home – may overlap with above)
- Severity/need
- Relationship with GP
- Not wanting to bother GP
- Fear of antidepressant prescription
- GP ability to do something about it
- Access barriers (time, continuity, gatekeeping)

Attitudes to mental health treatment (inc others’ experiences)

- General views around treatment
- General views around talking to someone (when no specific provider mentioned)
- Willingness to try (including vs nothing can help)
- Perceptions of psychiatry
- Perceptions of group talking therapies
- Perceptions of individual talking therapies
- Perceptions of antidepressants

Experiences of mental health treatments

- Decision-making (passive vs active role)
- Psychiatry
- Experiences of group talking therapies
- Experiences of individual talking therapies (code counselling vs CBT if possible)
- Previous experience of medication for mood

Other health care experiences

- Experience of physical health treatments
- Insomnia treatments
- “the system” – health (fairness, waiting times, lack of funds, reciprocity etc)
- Ageism in healthcare
- Support from long term condition providers that impacts on mental health (e.g. admiral nurses providing point of contact)

Community services

- Perceptions of social prescribing referrals from healthcare professionals
- “the system” – local authority and access
- Paid support (inc carers, cleaners etc)
- Support within sheltered housing environments
- Support groups (mental and physical health)
- Shared interest groups
- Social groups
- Other support services (e.g. Age UK)
- Others’ experiences of community services
- Transport

New service characteristics (including hypothetical and past experiences)

- Helpful components
- Unhelpful components
- Tailoring to person or situation
- Healthcare professional characteristics
- Use of technology for delivery
- One-to-one vs group
- Timing, duration and continuity
- How to access
