# Supplementary material for: Supporting frail older people with depression and anxiety: a qualitative study
Source: Aging Ment Health. 2019 Oct 16;24(12):1977–84. doi: 10.1080/13607863.2019.1647132 (PMC8842711; doi:10.1080/13607863.2019.1647132)
Supplement: Supplemental Material [file CAMH_A_1647132_SM8088.docx]

*Thank you for taking part in this study. We know that feeling or low or stressed can be common in later life, particularly when people find everyday activities more of a struggle, and this can lead to a lower quality of life and further physical difficulties. We would like to understand your experiences of mood and wellbeing in later life so we can develop better ways of providing support to people in future through the NHS or social care. If there are any questions you would prefer not to answer, please let me know and we can skip over it.*

**Mental health experiences in later life**

- What sort of things do you do in a **normal week**? (e.g. seeing friends, going for a walk)
- How are you **feeling in yourself** at the moment?
- Have you felt low **recently**? Can you tell me more about this?
- Have you felt anxious recently? Can you tell me more about this?
- **How often** do you feel this way?
- Have you been through periods where you have felt low or anxious earlier in your life [under 65]? Could you tell me about that?
- [If yes] How does that compare to how you feel now?

**Self-managing feelings of stress or depression [depending on main issue]**

- **When you feel low [worried], what do you do**?
- What do you think is the **most helpful** way to deal with feeling low [anxious]? What about the least helpful?
- Is this the same as when you feel anxious [low]?
- How do your **current health problems** affect your mood?
- Have you looked for any information regarding ways to cope when you felt low/worried?
  - If yes, what sources of information did you seek? (prompt for accessing information online)
  - If no, why not?
- [If applicable] How does your previous experience of feeling low or anxious affect how you deal with anxiety/feeling low now?
- How well do your previous coping mechanisms help you now? Are there ways of coping that you've used previously that you can no longer use now?
  - Prompts: Exercise? Socialising with others?
- Is feeling low or worried something you ever talk about with others?
  - If so, who?
- What effect do **family and friends** have upon your mood?
  - Explore intergenerational linkages and effects of extended family
  - Explore effects of loneliness and how management differs
- (If receiving care) what effect do your **carers** have on your mood?
- What effects do your activities/beliefs [specify from earlier conversation] have upon your mood?
  - Explore effects of religion (meaning and community/support)
  - Explore meaning of any projects/activities
- What effects do you current situation have on your mood?
  - Explore effects of finances (caring?)
- **In an ideal world, what would help you to cope better with your worries/low mood?**
- **What do you want out of life at this point in life?**
  - What do you think is holding you back from this?
  - What might help address this?
  - How could you imagine someone else being involved?

**Support from NHS services**

- Have you **spoken to anyone** e.g. your GP about feeling low/worried? What happened?
- If yes: What prompted you to seek support for your mood?
- If no: Are there any particular reasons why you haven’t spoken to anyone about this?
- If threshold: explore what does (e.g. ‘being desperate’, ‘black hole’) that mean to you?

General: Sometimes a GP or nurse can refer you on to another person to help support you with your mood e.g. a wellbeing service, counsellor or psychologist, a psychiatrist, social activities. Have you ever been referred to one of these services?

- How did you feel about being offered this service? [prompts: concerns, expectations, prepared to try it?]
- What were your experiences of this service?
- What was helpful? Unhelpful? [prompts: ease of use, relationship with therapist, setting, side effects, how did this affect your experiences?]

If need further prompting re **specific services**:

- Sometimes your GP will prescribe you tablets such as antidepressants when you are feeling low. What are your views about these? [prompt: ease of use, side effects]
- Sometimes your doctor [or social worker?] can refer you to a service where you can meet a group of people and do an activity e.g. gardening, or a befriending service. This is called social prescribing. What do you think about this? [prompt: appropriate for needs?]
- Some people can also be referred to talking therapies, where someone will ask them about their mood and help them develop ways to overcome feelings of stress or low mood in the future. What do you think about this sort of therapy? [prompt: appropriateness, setting, therapist]

*[Prompts*: opinions, experiences of, how did this help (or not), would you use again]

**Services could include:**

- **Phone support**
- **Support groups**
- **Support from peers**
- **Skype support**
- **Counselling**
- **CBT (coping skills for feeling low/when you can’t stop worrying)**
- **PST (solving everyday problems such as X that may be getting you down)**
- **Antidepressants**
- **Social groups**

**A new service**

- **Would you be willing to try anything (else) to help with your mood?**
- **Who** would you feel most comfortable talking to if you were feeling depressed?
  - Prompt: healthcare professional, family, friend, care worker
  - Specific prompts: GP, social worker, carer, nurse, friend, family member, family carer, occupational therapist, counsellor, psychologist, psychiatrist
  - Mental health specialist vs non-specialist person
- **Where** would be a good place to talk to someone?
- **How often** would you want to see them?
- What do you think about someone talking to you about your mood over the phone?
- What do you think about using online services to help cope with mood? e.g. a video conversation, skype or email, or reading about how people cope with your situation?
  - What would encourage you to use these online services? (e.g. someone by your side to demo how to operate, user-friendly interface)
  - If not, why not?
- If we developed a new service, should it be focussed only on mood or on **other things** too? Like what? [prompts: socialising, activities]
- What would make you **more likely to use** a service like this? Less likely?
- Would it be helpful for anyone else to be involved e.g. friend, relative? In what way?
- How could we ensure people would use this service?

Other prompts

- Do you know **anyone else who has felt this way**? How does it affect them? What do you think would help?

*Thank you for taking part in this study. We will add your interview to others we’ve already collected, which will help us improve and develop services for low mood and anxiety in future.*

Voucher + list of services
